# Functional and computational identification of a rescue mutation near the active site of an mRNA methyltransferase

**DOI:** 10.1038/s41598-020-79026-2

**Published:** 2020-12-14

**Authors:** Pierre-Yves Colin, Paul A. Dalby

**Affiliations:** Department of Biochemical Engineering, University College London, London, WC1H 0AH UK

**Keywords:** Transferases, Molecular modelling, Biocatalysis

## Abstract

RNA-based drugs are an emerging class of therapeutics combining the immense potential of DNA gene-therapy with the absence of genome integration-associated risks. While the synthesis of such molecules is feasible, large scale in vitro production of humanised mRNA remains a biochemical and economical challenge. Human mRNAs possess two post-transcriptional modifications at their 5′ end: an inverted methylated guanosine and a unique 2′O-methylation on the ribose of the penultimate nucleotide. One strategy to precisely methylate the 2′ oxygen is to use viral mRNA methyltransferases that have evolved to escape the host’s cell immunity response following virus infection. However, these enzymes are ill-adapted to industrial processes and suffer from low turnovers. We have investigated the effects of homologous and orthologous active-site mutations on both stability and transferase activity, and identified new functional motifs in the interaction network surrounding the catalytic lysine. Our findings suggest that despite their low catalytic efficiency, the active-sites of viral mRNA methyltransferases have low mutational plasticity, while mutations in a defined third shell around the active site have strong effects on folding, stability and activity in the variant enzymes, mostly via network-mediated effects.

## Introduction

Nucleic acid-encoded drugs provide an economical solution to the development and manufacturing of new therapies. Among them, messenger RNAs (mRNAs) have gained recent attention since, unlike DNA-based therapies, mRNAs enable the delivery of genetic information without the risk of genome integration. mRNAs for new vaccines^1,2^ as well as for the expression of therapeutic proteins after cell delivery^3^ have been demonstrated and entered clinical development^4^. mRNAs are post-transcriptionally modified at their 5′ (cap) and 3′ ends (poly A tail), improving both the initiation of the translation and the stability in eukaryotes. The 5′ cap (cap 0) is formed of an N7-methylated inverted guanosine (m^7^G) linked to the first transcribed nucleotide by a triphosphate linker. In higher eukaryotes, the cap 0 is further methylated at the 2′oxygen of the ribose on the first nucleotide making up a structure called cap 1^5^. Members of the large double-stranded DNA poxvirus family have evolved the ability to methylate the first transcribed nucleotide of their mRNA, probably as a strategy to escape immunogenic response in the host cell^6,7^. By analogy, therapeutic mRNAs must be non-immunogenic in order to restore or supplement the function of altered genes by mRNA-based therapy.

The enzyme responsible for the 2′O-mRNA methyltransferase activity in the poxvirus Vaccinia is VP39^8^, a bifunctional protein that also acts as a processive factor when in complex with VP55, an mRNA poly(A) polymerase^9^. Unlike the orthologous proteins from the PFAM^10^ PARP-regulatory protein family (PF01358), the structure of the monomeric, apo^11^ and holo^12^ forms) and the heterodimeric^13^ forms of VP39 have been determined. The structure is characterised by a Rossmann fold in the core of the protein^11^.

VP39 catalyses the transfer of a methyl group from a molecule of s-adenosylmethionine (SAM) onto the 2′ oxygen of the first transcribed nucleotide of the cap 0 mRNA following a random bi-reactant mechanism^8^. The human 2′O-mRNA methyltransferase CMTr1 is a member of the same superfamily (PFAM Clan 063) whose structure has been determined. CMTr1 differs from VP39 in the mRNA binding mechanism and, unlike VP39^14^, CMTr1′s activity is not m^7^G-dependent^15^. For this reason and because VP39 is the most characterised viral mRNA 2′O-methyltransferase to date, it is a potential candidate for the in vitro post-transcriptional enzymatic methylation of therapeutic mRNAs in an industrial context.

The catalytic efficiency of the mRNA methyltransferase activity of VP39 is low, on the order of 10^–3^ s^-1^ M^-1^, corresponding to only 1–7 turnovers under the conditions of previously reported assays^16^*.* The structural determinants for the mechanism of VP39 are not yet fully understood, and so it is not clear whether the activity of the enzyme is already limited by evolution, or by the inherent combined reactivity of the substrate and cofactor. Understanding the role of the active-site residues in their mechanism and their mutability will be crucial in informing the design, or directed evolution, of new variants that are better targeted to novel substrates.

Extensive alanine scanning has been conducted previously to identify functional residues involved in methyltransferase activity^17^. Lysine 175 (K175) is fully conserved in the PFAM PF01358 family and is the key residue for the methyltransferase activity^11^. Two residues: Aspartate 138 (D138) and Arginine 209 (R209) are largely conserved in the PFAM PF01358 family and likely to participate in the activation of K175^11^ . An orbital steering mechanism has been proposed implying that K175 must be in its deprotonated form to accept the RNA 2′OH proton during methyl transfer^18^. In agreement with a deprotonated Lysine, the pK_a_ of K175 was found to be shifted down to 8.5^19^, not far from the measured pH-optimum for VP39 of 7.5 (Lockless, S. W.; Cheng, H. T) or 8^8^. Density Functional Theory (DFT) studies of the transfer of a methyl group from a sulfur onto a hydroxyl, further confirmed that a proton acceptor significantly lowered the high activation energy barrier of the methyltransfer reaction, eventually matching the experimental *k*_cat_^20^.

While an alanine scanning approach is useful in identifying the potential role of active-site residues, it is unlikely to identify functional mutations or reveal any compensatory interactions between residues. In the absence of a high throughput screening/selection necessary for testing a high number of enzyme variants, we reasoned that functional mutations could be identified in homologous and orthologous sequences found in the PF01358 family, thus testing only a low number of enzyme variants. We directly targeted the residues surrounding the catalytic lysine K175, as the most likely to modify the electrostatics in the active site, and hence the pK_a_ of K175, with the potential of influencing the methyltransferase activity.

## Results

### Residues around the catalytic lysine are highly conserved in the PARP regulatory PFAM family

**Figure 1:**
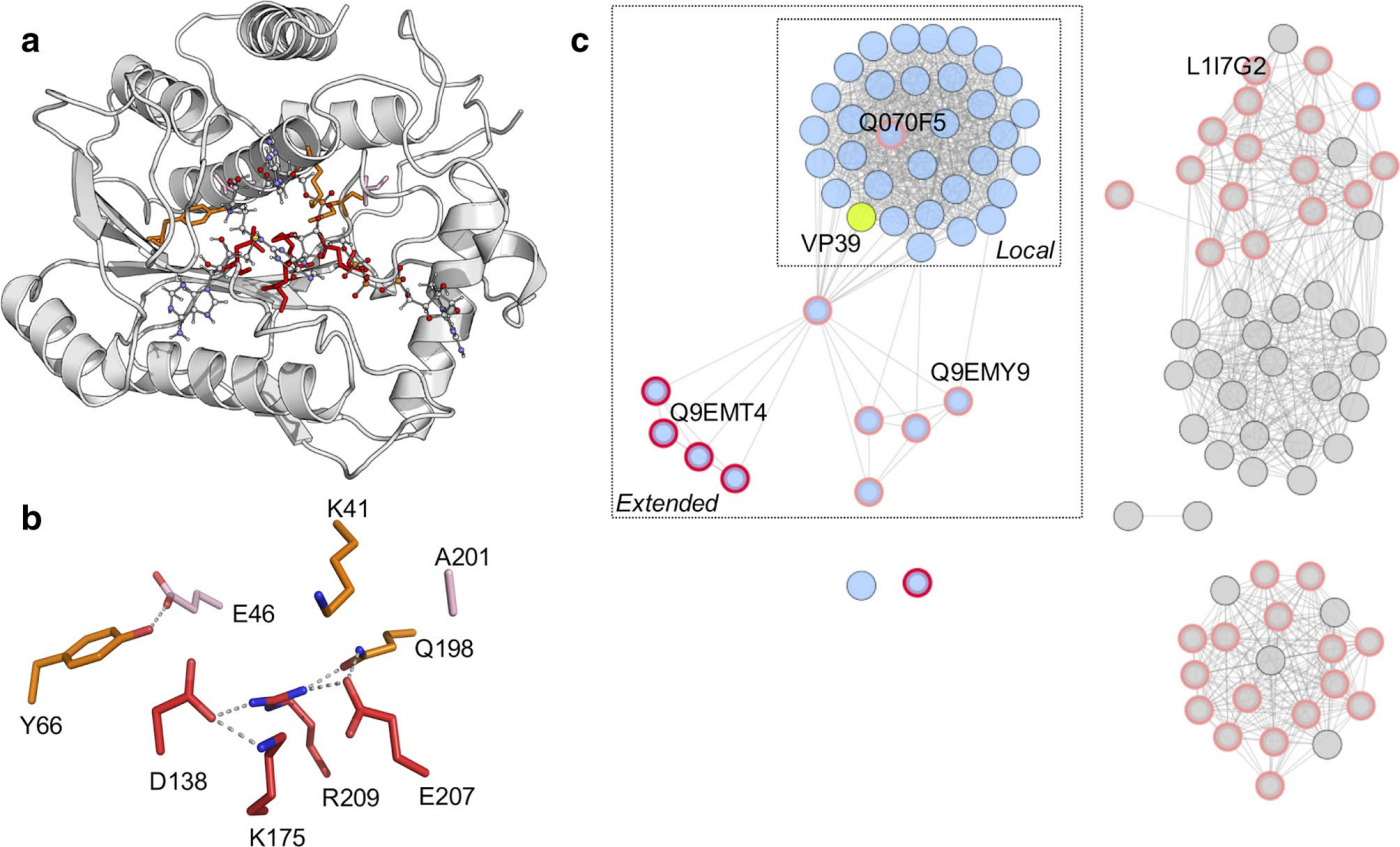
The active site of the 2′O-mRNA methyltransferase VP39 is mostly conserved within the PFAM PF13058 family. (**a**) structure of the 2′O-mRNA methyltransferase VP39 (1av6^12^) in complex with cap 0 mRNA and s-adenosylhomocysteine. Coloured residues are residues from the 1st (red), 2nd (orange) and 3rd (pink) shells. (**b**) Active site of the 2′O-mRNA methyltransferase VP39. Residues forming the 1st shell, 2nd and 3rd shell are represented in red, orange and pink, respectively. (**c**) Sequence-similarity network representing the whole PFAM PF01385 family comprising 2′O-mRNA methyltransferases. Each node in the network shows one of the 106 sequences described within the family (in May 2020). Edge length represents sequence similarity at the amino acid level. The similarity is represented by an edge when the alignment scores an E-value lower than 1.e^−30^, corresponding to a median pairwise sequence identity of 32.9% over more than 250 residues. The sequence of VP39 is represented by a yellow circle. Sequences identified in viral genomes are shown in blue, sequences identified in Trypanosomes and other eukaryotes are shown in grey. Nodes with red circles correspond to sequences with an Aspartate residue at the position corresponding to K41 in VP39. Nodes with a mutation corresponding to the position 201 in VP39 are circled in pink. Q070F5 (from the Nile *crocodilepox* virus), L1I7G2 (from *Guillardia theta*) and Q9EMT4 and Q9EMY9 (from *Amsacta moorei* entomopoxvirus) are highlighted in the network. The ‘local’ region of the SSN corresponds to sequences with a median pairwise sequence identity of 56%. The ‘extended’ sequences are connected to the ‘local’ region via the sequence Q9YW51, only sharing a median pairwise sequence identity of 33% with 15 sequences of the ‘local’ group. Logo plots for the probability of the residue identities at the positions corresponding to the region of interest in VP39 found in sequences of the ‘Local’ region in the SSN, the ‘extended’ region of the SSN and in the entire PF0138 family are represented in Supplementary Fig. 2.

The active site in VP39 is centred around K175, which activates the 2′-hydroxyl group of the first transcribed nucleotide, for nucleophilic attack on the methyl group, by a mechanism of orbital steering^18^. Several residues form a highly dense hydrogen-bond network (Fig. 1a,b) around K175.

**Figure 2:**
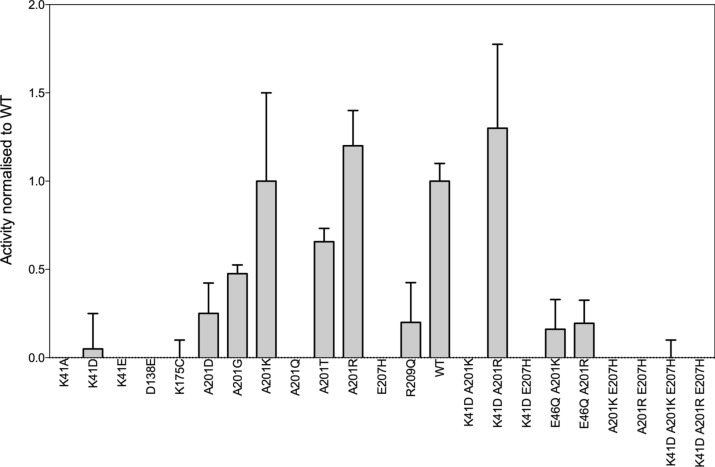
Methyltransferase activity of all the variants tested in this study. The activity was measured using an end-point assay quantifying the concentration of s-adenosylhomocysteine after 2 h at 37 °C in Tris–HCl 50 mM, MgCl_2_ 30 mM, 25 mM ATP as described in the Methods. Activities were measured in triplicates or duplicates (K41D, K41E, D138E, E207H) in at least 2 independent experiments. Data represent median values normalised to the WT activity. Error bars represent interquartile range of the activity distributions. All values and standard deviations are reported in the Supplementary Table 1.

A first shell of residues was defined as the three residues within 4 Å of K175 (Fig. 1b): D138, R209—previously identified as part of the catalytic triad^19^, and E207. D138 is involved in activating K175^19^ as well as interacting with the amino group of s-adenosylmethionine. The steric mutation D138E was enough to drop the activity of the variant below the detection limit of our methyltransferase assay (i.e. corresponding to the signal measured with the known knock-out mutant K175C) (Fig. 2), underlining the importance of the precise positioning of the carboxylate group of this residue. We reasoned that potential functional mutations could be identified in orthologous and homologous sequences in the PFAM family PF01358. We constructed a sequence-similarity network^21^ (SSN) that mapped all the 106 sequences of the family as a function of their pairwise sequence relationships (Fig. 1c). From this network, we derived three groups of sequences in the family: i. the ‘local’ sequences corresponding to orthologous sequences the most identical to VP39, ii. the ‘extended’ sequences comprising additional more distant viral sequences and iii. the rest of the family mostly comprising homologous sequences from Eukaryotes (Fig. 1c). The residues of the first shell are almost fully conserved throughout the family, yet one mutation E207H was identified in five sequences of the ‘extended’ sequences (Fig. 1c). Another mutation R209Q was identified in the L1I7G2 variant from a *Geminigeraceae* organism. Despite being identified in homologs with low sequence homology to VP39 (25–27% and 30% sequence identity for the sequences bearing the mutations E207H and R209Q, respectively), both mutations were constructed in VP39. As expected, the two mutations were highly detrimental when the variants were tested for methyltransferase activity (Fig. 2 and Supplementary Table 1).

### Functional third-shell mutations could only be identified in close homologs

It was previously hypothesised that K41 may be important for lowering the p*K*_a_ of the RNA 2′hydroxyl^12^, potentially participating in mRNA binding^22^, and was described as part of the K-D-K motif found in other methyltransferases^22^. This residue is largely conserved among the PF01358 family except in five orthologous sequences found in the ‘extended’ viral sequences (shown with red circle on Fig. 1c). Indeed, a previous multiple sequence alignment^23^ revealed that Q9EMT4 from *Amsacta moorei* entomopoxvirus, and four other sequences (red circles in Fig. 1c), displayed an Aspartate residue at this position, and was further confirmed using structural alignment between the structure of VP39 (1av6^12^) and a model structure of Q9EMT4 (Supplementary Fig. 1). Although part of the PF13058 family, it is not clear whether Q9EMT4 even has any methyltransferase function, despite being essential for the virus’ replication. *Amsacta moorei* entomopoxvirus indeed possesses another gene (yet non-essential), encoding for an active mRNA methyltransferase^23^ . While most sequenced viruses have methyltransferases that are close orthologs to VP39 (i.e. > 50% sequence identity with VP39 and grouped in the ‘local’ network in Fig. 1c), *Amsacta moorei* entomopoxvirus had two distant orthologs to VP39 in the PF01358 family, suggesting a different mechanism in the 5′ cap maturation. The orthologous mutation K41D was nonetheless tested in VP39 as well as the longer side chain K41E. Both charge-inversion mutations were detrimental for the transferase activity, whereby the activity of K41E dropped below the assay detection limit, whereas the activity of K41D was just above the lower limit, with a median methyltransferase activity of around 5% that of WT (Fig. 2). As a control, we also constructed and tested the K41A mutation: the alanine mutation was found to be deleterious (Fig. 2), further suggesting that a basic residue was preferred at this position in VP39.

The side-chain of lysine 41 (N_ζ_) was within 4 Å of atoms from the side-chains of four residues in VP39 (1vp3^11^): L42 (K41′s direct neighbour residue), E207, Q198 and A201 (Fig. 1b). Of these, position 201 was the only residue not fully conserved (supplementary Fig. 2) in the closest virus orthologs constituting the ‘local’ sequences in Fig. 1c. Ortholog Q070F5 from the Nile crocodile poxvirus possesses an arginine instead of an alanine at this position (Fig. 1c and Supplementary Fig. 2). When analysing the more distant orthologs (‘extended’ sequences in Fig. 1c and sequences from Trypanosomes and other Eukaryotes, in grey on Fig. 1c), it appeared that the residue corresponding to position 201 in VP39 was weakly conserved (Supplementary Fig. 2). Inspired by the mutations found in orthologs at the equivalent position, a 6-membered library of VP39 variants was built comprising the following mutations A201T, A201G, A201Q, A201D, A201K and A201R. Both the polar glutamine (A201Q) and the charge inversion A201D mutant completely abolished the activity . The A201G and A201T mutations were found to be less detrimental, the median activity was found to be 51% and 60% that of WT activity, for A201G and A201T respectively (Fig. 2). However, neither of the basic mutations (A201K and A201R) led to a significant drop in the methyltransferase activity. In fact, the median activity was similar to WT for A201K, and even 1.2-fold higher than WT for A201R (Fig. 2). Thus, the addition of a potential positive charge in the third shell of the active site (as defined in Fig. 1c) appeared neutral—and even slightly beneficial for A201R—as measured in our functional assay The basic mutation A201R was identified in the closest homolog amongst those with a different residue at position 201 (Q070F5, with > 52% sequence identity to VP39) whereas the least active A201 variant bore the mutation A201Q, identified in the homologous sequence with a very low sequence identity to VP39 (Q9EMY9, < 25% sequence identity to VP39). Overall, the “acceptability” of a homologous mutation at position 201, determined by the level of activity compared to WT, seemed to be dependent on how similar the orthologous sequence was to the sequence of VP39 (Supplementary Fig. 4). More specifically, a comparison of the residues directly surrounding residue 201 in the different homologs suggested a local context-dependence on the identity of residue 201 (Supplementary Table 2). As an example, the absence of proline residues around the equivalent residue 201 in the ortholog Q9EMY9 (Supplementary Table 2) may enable different conformations of the loop comprising glutamine 201, thus permitting the A201Q mutation. For this reason, in order to investigate local context dependencies on the mutations A201K and A201R mutations and inspired by the sequences from the ‘extended’ group of orthologous sequences (Fig. 1c, supplementary Figs. 1, 2), a serie of 2nd generation mutants (double and triple mutants) were constructed.

### Context-dependence of the effects of two basic mutations in the third shell

The positions that were investigated in the 2nd generation mutants were selected as a function of two parameters: *i.* being polar residues and/or *ii.* with side chains positioned in direct or indirect contact with the first shell residues (K175, D138, E207 and R209) that were conserved in the ‘local’ sequences of the SSN but were diversified into polar residues at the equivalent position in the ‘extended’ sequences of the SSN (Fig. 1c and Supplementary Fig. 2). Based on these criteria, the positions investigated in the 2nd generation mutants corresponded to the residues K41, E46, A201 and E207. Seven double-mutants and two triple-mutants were purified under similar conditions, and their methyltransferase activity was tested.

The mutations A201K and A201R had dramatically opposite effects in second-generation mutants: K41D-A201K was inactive, whereas A201R not only rescued the methyltransferase activity of the inactive K41D variant, but increased it to a median activity 1.7-fold higher than WT (Fig. 2). A partially charged histidine at position 207 was originally hypothesised to destabilise the protonation state of lysine 175 through repulsion effects. However, none of the mutants bearing the mutation E207H were active (Fig. 2), emphasising the importance of the glutamate residue at position 207 in the different contexts tested. Yet, when comparing production yields following small-scale fermentation and affinity chromatography, a clear drop in protein yield was observed for the double mutant A201K-E207H and triple mutant K41D-A201K-E207H (Supplementary Fig. 5). Replacing the lysine by an arginine at position 201 improved the production yield in both cases (Supplementary Fig. 5). This observation suggested that some 2^nd^ generation mutations had effects on the protein expression and folding, leading us to further investigate the stability of all variants: single mutants and 2nd generation mutants.

Intrinsic tryptophan fluorescence of all variants was measured over a temperature ramp to i) measure their melting temperatures (*T*_m1_) and ii) measure the variants’ tryptophan red-shift values upon unfolding. Analysis of the difference of fluorescence barycentric means upon protein unfolding enabled to distinguish variants with different folding-unfolding transitions. Since none of the tryptophans were targeted by site-directed mutagenesis in this study, the fluorescence barycentric means of the unfolded states was similar for all variants. Thus tryptophan red-shift values were only dependent on the fluorescence barycentric means of the folded state of each variant and was, therefore, used as a proxy to group variants with similarly folded states.

**Figure 3:**
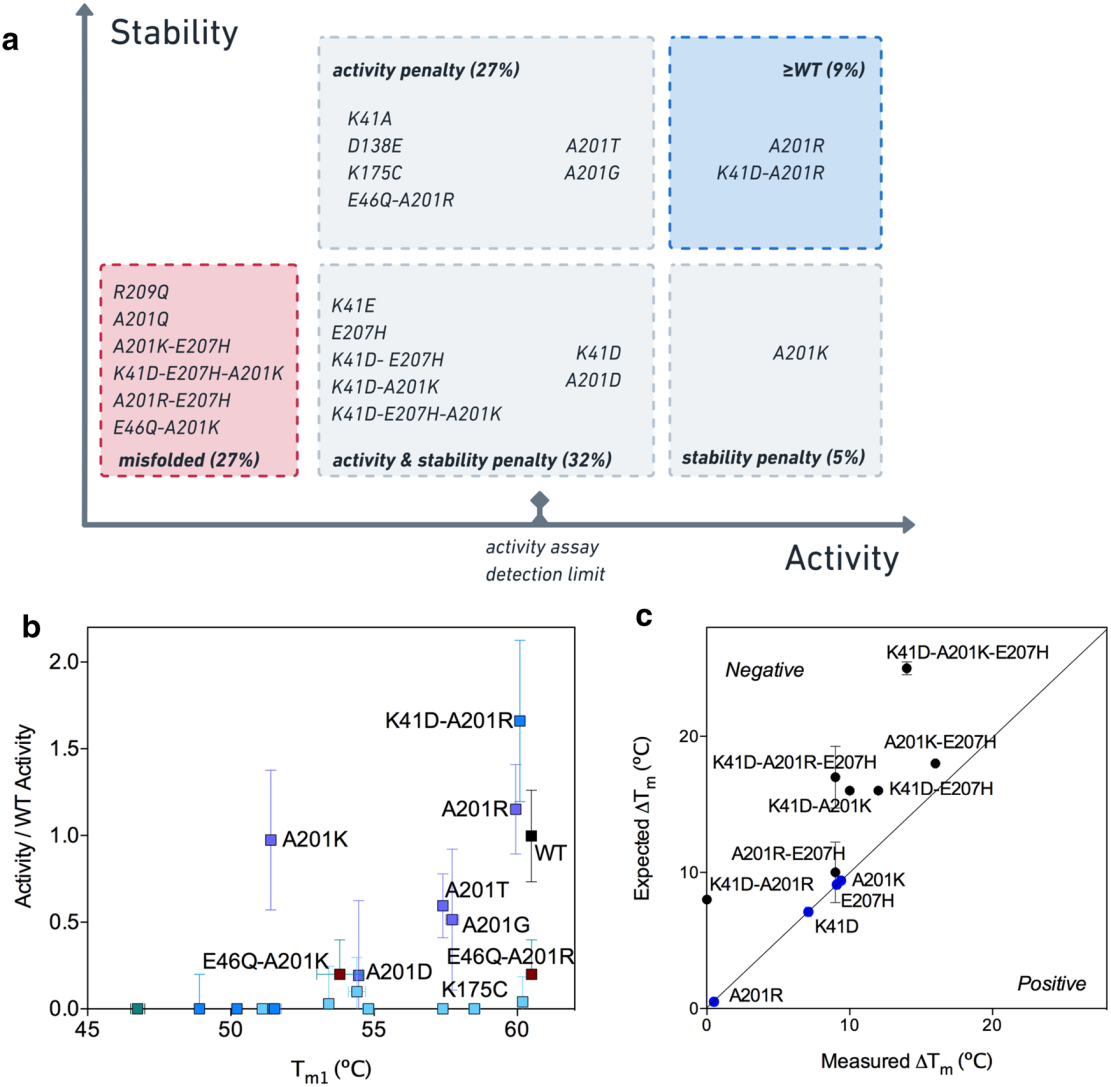
Activity-stability trade-off in single and 2nd generation methyltransferase mutants. (**a**) Grouping mutants as a function of folded-states, stability and transferase activity. The top right blue panel contains variants with WT-like stability and activity. The lower panels contain mutants with T_m1_ > 5 °C lower than WT. The mutants in the activity penalty panels but above the ‘activity assay detection limit’ are variants with transferase activity lower than 60% of WT activity. The variants in the lower left red panel appeared as misfolded in the analysis of their tryptophan fluorescence barycentric mean shift upon unfolding (Supplementary Fig. 4). (**b**) Activity ratios (Fig. 2) plotted against the first transition point (T_m1_) of the melting curves. WT is in black, single mutants are in light blue, single A201 mutants are in purple, double mutants are in dark blue, E46Q mutants in brown and triple mutants in green. (**c**) Negative epistasis between second and third shell residues. ΔT_m_ is the variation between the first transition points in the melting curves of WT and mutant. The expected ΔT_m_ corresponds to the ΔT_m_ value if the effects of mutations are purely additive. Any deviation from the diagonal line suggests epistatic interactions between the mutations. All T_m_ and ΔT_m_ values are reported in the Supplementary Table 1.

Most variants including VP39 WT had a barycentric mean fluorescence shifted by 7.8 nm (± 0.4) upon unfolding, suggesting that they were similarly folded (Supplementary Fig. 4). Some variants yielded red-shift values lower than 6 nm upon unfolding, suggesting misfolded states. These variants included A201K-E207H and K41D-E207H-A201K, also corresponding to variants with low expression yields and A201R-E207H, E46Q-A201K, R209Q and A201Q, corresponding to variants with lower thermal stability than WT VP39 (*T*_m1_ > 5 ºC below WT’s *T*_m1_—Supplementary Table 1 and Supplementary Fig. 6). Based on these observations, we concluded that this first group of mutants were most likely inactive transferases due to their misfolded state, and so not necessarily due to active-site effects (Fig. 3a).

**Figure 4:**
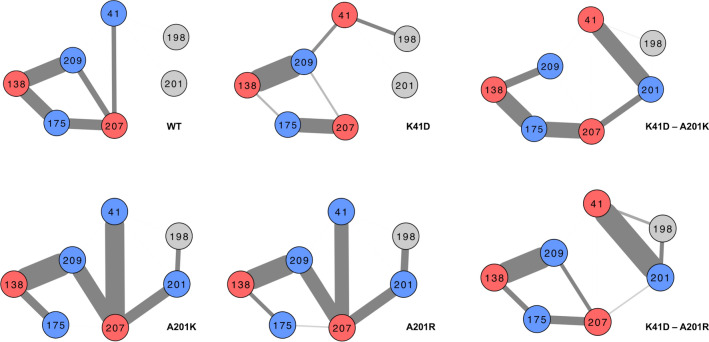
Hydrogen-bonds rearrangements in the active site of 2nd and 3rd shell mutants in absence of mRNA. The diagrams show hydrogen-bonds occurrence between residues in the 1st, 2nd and 3rd shell along molecular dynamics trajectories of the apo forms of the different variants. The occurrence (i.e. evaluated by a number of true events in frames along molecular dynamics trajectories) of having a hydrogen-bond between two residues’ side chains is represented by the thickness of the edge connecting two nodes. The thicker the edge is, the more often a hydrogen-bond is observed between the two linked residues in the frames. > 3000 frames were analysed from three independent (10 and 30 ns) trajectories.

A second group of variants appeared to be properly folded on the basis of expression yield and unfolding red-shift values (Supplementary Figs. 5 and 6), yet were inactive methyltransferases under our assay conditions (Fig. 2 and Fig. 3a). Within this group, it appeared important to distinguish between inactive transferase yet folded and stable variants (K41A, D138E, K175C and E46Q-A201R), from inactive and poorly stable variants (K41E, E207H, K41D-E207H, K41D-A201K and K41D-E207H-A201R) (Fig. 3a). Inactive but stable variants were those bearing mutations in the previously described K-D-K methyltransferase motif^27^ and were expected to bear mutations specifically affecting transferase efficiency. In this sub-group was also found the variant E46Q-A201R which clearly differed from the misfolded E46Q-A201K variant, highlighting the stabilising role of A201R in this context (Supplementary Fig. 2). However, the E46Q mutation clearly inactivated the transferase activity in the presence of A201K or A201R. Structure analysis of VP39 WT revealed that E46 was deeply buried within the protein core interacting with Y66, in direct contact with D138 (Fig. 1b) and is highly conserved throughout the PFAM PF01385 family but in the sequences found in the ‘extended’ network from the SSN (Fig. 1c, Supplementary Fig. 2). Our results confirmed that a functional glutamine at the equivalent position 46 was context-dependent.

**Figure 5:**
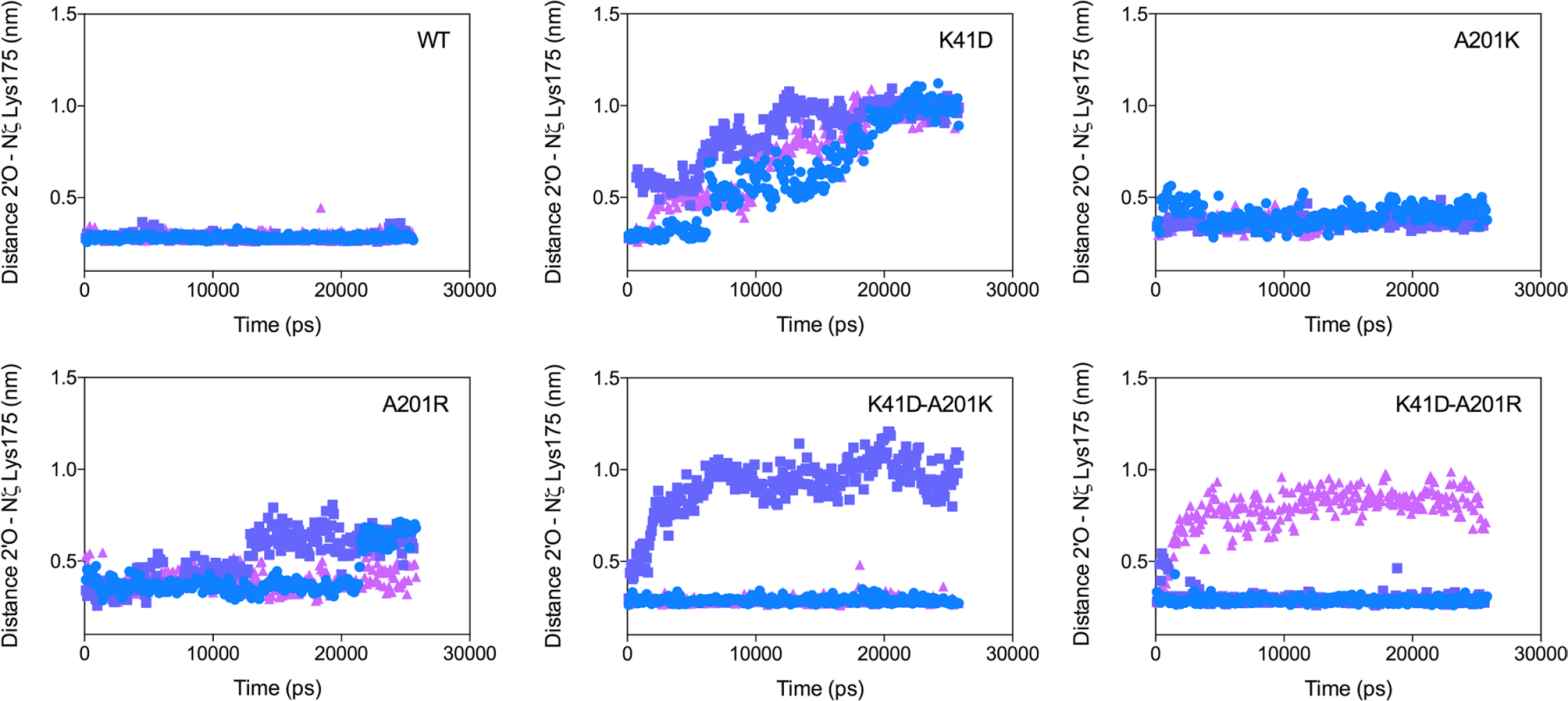
A201K and A201R mutations improved mRNA positioning in the K41D background. Distance between the oxygen atom at the position 2′ in the first transcribed nucleotide and the nitrogen atom ζ of the Lysine 175 measured along 25,000 ps-long trajectories on the six variants WT, K41D, A201K, A201R, K41D-A201K and K41D-A201R. Blue circles, violet squares and pink triangles correspond to replicates of three independent trajectories. All the simulations were performed with a protonated form of the lysine 175.

**Figure 6:**
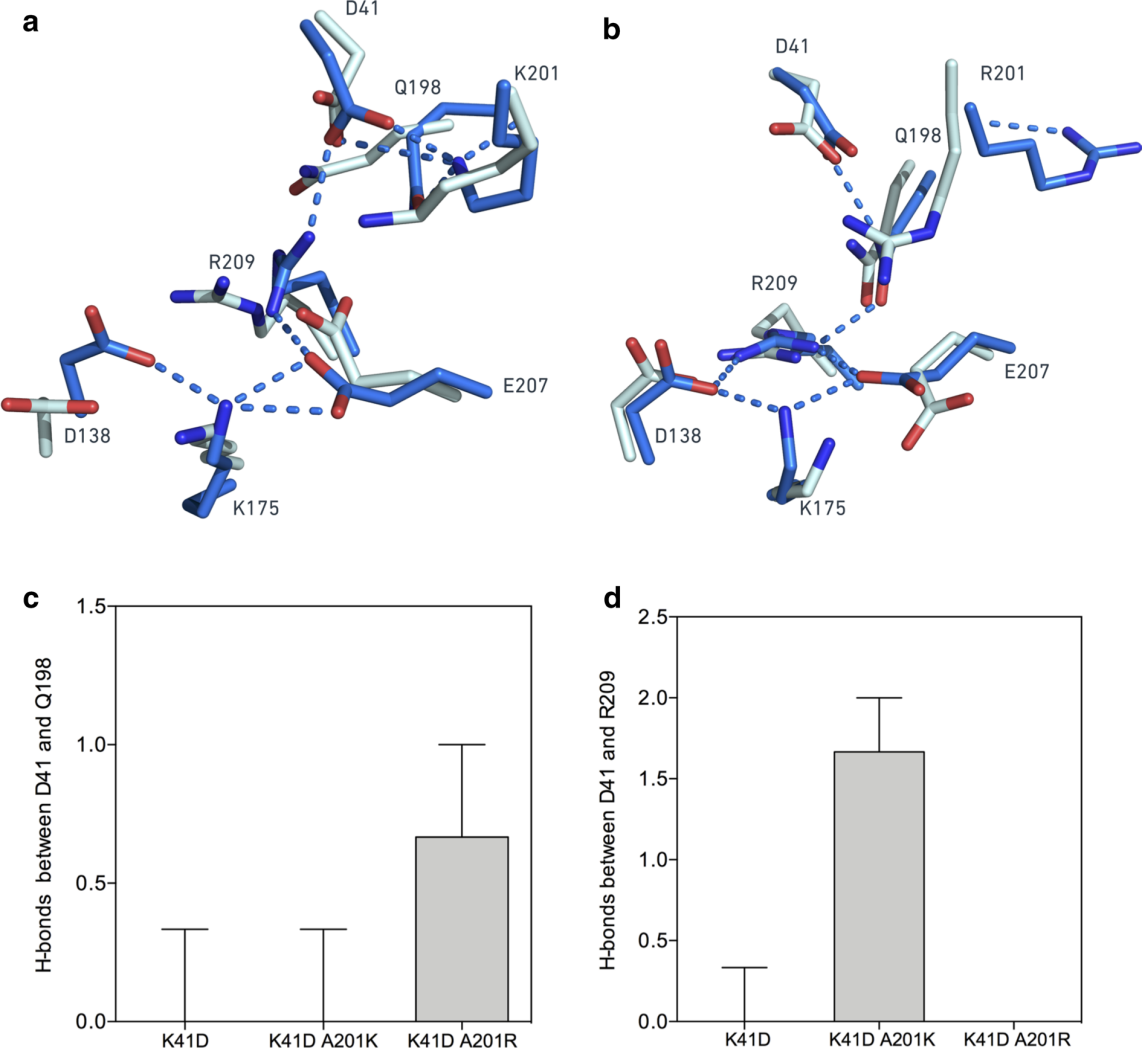
Opposite effects of arginine and lysine residues at position 201 on the first shell integrity in the presence of mRNA. MD simulations in the presence of mRNA revealed different conformations of the 1st, 2nd and 3rd shell residues’ side chains between the variants K41D-A201K and K41D-A201R. (**a**) Superimposition of the active sites of the variant K41D-A201K in absence of mRNA (cyan) and presence of mRNA (blue). In presence of mRNA, the residue D41 is shown hydrogen-bonded to K201 and R209, inducing a displacement of R209, disrupting the hydrogen-bond network in the 1st shell of the active site. (**b**) Superimposition of the active sites of the variant K41D-A201R in absence of mRNA (cyan) and presence of mRNA (blue). The residue R201 is pointing outward from the active site, enabling Q198 to interact with D41. The hydrogen-bond network in the 1st shell of the active site is maintained. (**c**) Median number of hydrogen-bonds between the residues D41 and Q198 along 25 ns trajectories (performed in triplicates) in the three variants WT, K41D-A201K and K41D-A201R. (**d**) Median number of hydrogen-bonds between the residues D41 and R209 along 25 ns trajectories (performed in triplicates) in the three variants WT, K41D A201K and K41D A201R. Error bars represent the interquartile of the hydrogen-bond distributions.

A third group of variants was composed of enzymes endowed with different levels of methyltransferase activity. Overall, the melting temperatures (*T*_m1_) of the active variants correlated well with their methyltransferase activity (Fig. 3b) but we can distinguish three sub-groups of mutations: (i) The A201 mutations with transferase activities that correlated with their first melting-transition temperatures (Fig. 3b), suggesting that activity was a function of kinetic unfolding or aggregation rates prior to assays; (ii) A201K with high transferase activity, yet poor thermostability; and (iii) A201R and K41D-A201R with retained stability and activity.

Analysis of the independent contributions towards activity and stability for each mutation in the different background sequences, clearly highlighted the opposing effects of the A201K and A201R mutations. A201R had a stabilising effect upon K41D and K41D-E207H, which both started with low stability. Its contribution to stability appeared to be neutral in the context of WT or K41D-A207H backgrounds, while A201K was deleterious in all cases (Fig. 3a). Further to this, the individual contributions of the mutations K41D, A201R and A201K, revealed clear negative epistatic interactions between the residues at position 41 and 201 (Fig. 3c). Overall, A201R appeared stabilising whereas A201K appeared destabilising, but at this stage it remained unclear why the effects of these two basic residues on stability and activity, were context-dependent or so clearly different. To understand at the molecular level the contribution and interplay between positions 41 and 201 on the stability and activity of the variants, we performed molecular dynamics (MD) simulations on the following six variants: WT, K41D, A201K, A201R, K41D-A201K and K41D-A201R.

### MD analysis of the compensatory mechanism of A201R in the K41D background

Our experimental data suggested that the replacement of K41 by a negatively charged residue (K41D mutation), was detrimental to the thermostability as well as for the methyltransferase function. We hypothesised that local rearrangements of the hydrogen-bond network in the active site of the enzyme variants could have an effect on the overall fold stability. We tested this hypothesis by measuring the occurrence of hydrogen-bonds between the residues of the active site of six enzyme variants: WT, K41D, A201K, A201R, K41D-A201K and K41D-A201R using > 3,000 structure snapshots captured along 30 ns MD trajectories. The analysis of the hydrogen-bond networks showed that the K41D mutation abolished the interaction between residue 207 and 41, and disrupted the overall hydrogen-bonding network between D138, R209, K175 and E207 (Fig. 4). Introducing a basic residue at position 201 also affected the same hydrogen-bonding network, in a residue-dependent manner. The A201K mutation completely abolished the interaction between K175 and E207, whereas this interaction was retained in presence of A201R (Fig. 4). Furthermore, the interaction between residues 201 and 41, was retained more frequently in the double mutant K41D-A201R, probably due to a new salt bridge between the D41 and R201 residues, than it was in the double mutant K41D-A201K (Fig. 4). Overall, the maintenance of a dense hydrogen-bonding network between D138, R209, K175 and E207, partially correlated with the experimentally determined retention of high stability (measured by Tm, Fig. 3). We further hypothesised that a more frequent interaction between D41 and R201 in the double mutant K41D-A201R was more efficiently able to mask the destabilising effect of the negative charge introduced by K41D. Indeed**,** the presence of a negative charge at position 41 shifted the median value of the pK_a_ of lysine and arginine 201 in the double mutants towards alkaline values, further emphasising the electrostatic impact of the K41D mutation (Supplementary Fig. 7).

**Figure 7:**
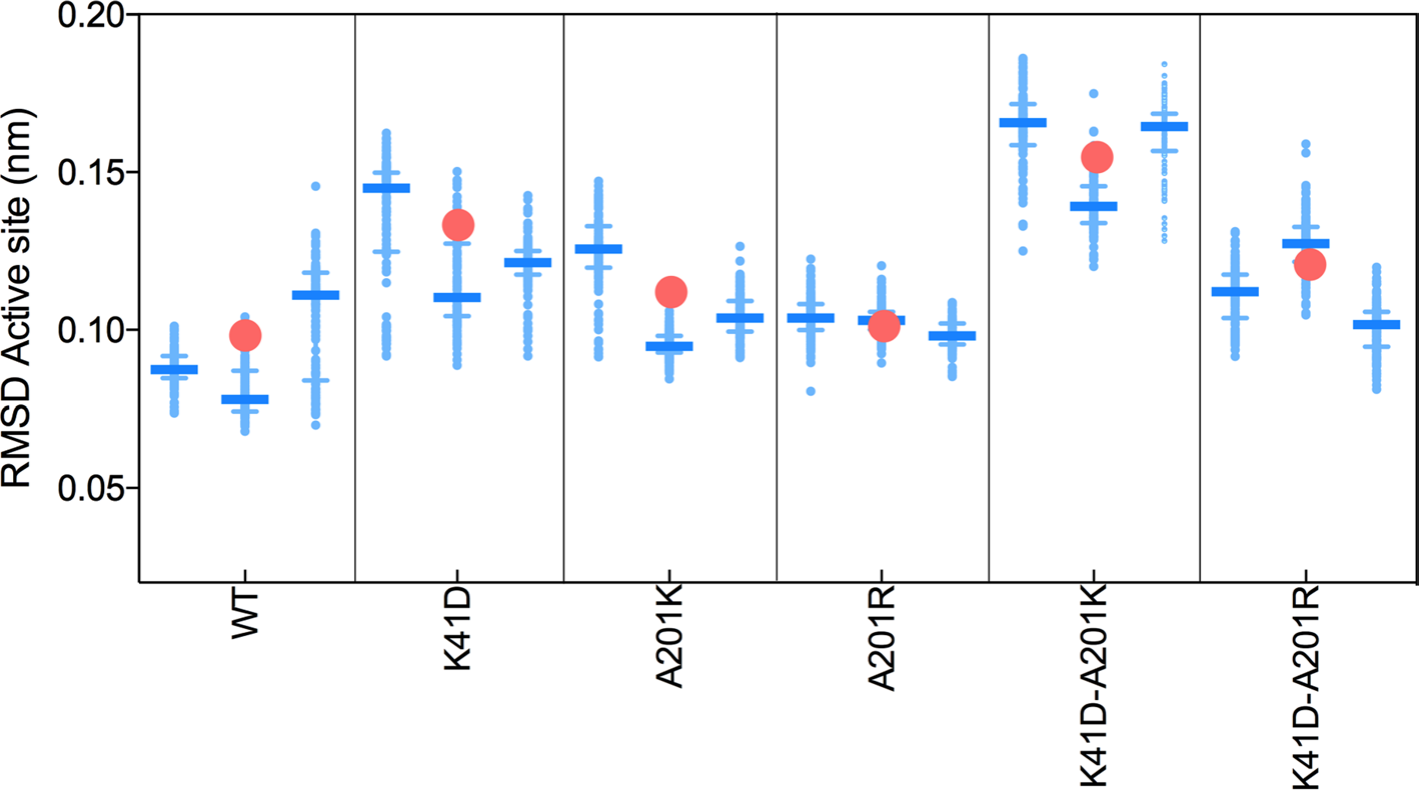
Rigidity in the first shell of the active site in the presence of mRNA. The Root-mean-square deviation (RMSD) was calculated using > 500 frames extracted from three independent 25 ns-trajectories every 100 ps. The light blue dots represent all RMSD values and the blue horizontal bars represent the median of the distribution (with the interquartile represented by the error bars). A low RMSD is interpreted as a higher rigidity. A higher rigidity was observed in the single variants that were catalytically active: with an median RMSD across three trajectories (red circles) of 0.090, 0.104 and 0.101 nm for WT, A201K and A201R, respectively, as opposed to 0.130 nm for K41D. The double mutant K41D-A201K has the most flexible active site (median RMSD = 0.157 nm). Replacing the lysine by an arginine in the K41D-A201R rigidifies the active site (median of the three trajectories = 0.124 nm).

In order to understand the role of the different mutations on the transferase activity, new MD simulations were performed in the presence of cap 0 mRNA. VP39 is a random bi-reactant enzyme, i.e. the binding of s-adenosylmethionine does not influence the binding of cap 0 mRNA^8^, the MD simulations were therefore performed in presence of the cap 0 mRNA alone. . A key step in the catalytic mechanism of VP39 is to activate the 2′oxygen (2′O) of the second nucleotide in the mRNA for nucleophilic attack on the methyl group of the second substrate. The distance between the final nitrogen on the side chain of lysine 175 (N_ζ_) and the 2′O of the second nucleotide of the mRNA is therefore an important characteristic of active methyltransferases. We reasoned that this distance must be in the order of hydrogen-bond distance (~ 0.3 nm) in order to promote proton exchange. The N_ζ_—2′O distance was therefore measured as a function of time along MD trajectories performed on the six variants WT, K41D, A201K, A201R, K41D-A201K and K41D-A201R in presence of the mRNA substrate (Fig. 5). For all active single mutants tested, the N_ζ_—2′O distance was found to be 0.27–0.3 nm along the whole trajectory in the triplicates (Fig. 5), whereas the N_ζ_—2′O distance increased to up to 1 nm in the non-active single mutant K41D in the three replicate trajectories (Fig. 5), suggesting that this mutation decreased the binding affinity to the mRNA, or at least displaced the position of the mRNA from optimal activation of its 2′O by K175. By comparison, the N_ζ_—2′O distance was restored to values around 0.3 nm, in two out of three replicate trajectories in the K41D-A201K and K41D-A201R variants, suggesting that both the A201K and A201R mutations could mitigate the detrimental effect that the negative charge at position 41 induced on he N_ζ_—2′O distance (Fig. 5).

While the simulations in the absence of mRNA suggested more frequent interactions between R201 and D41, than between K201 and D41 (Fig. 4) in the double mutants, this did not explain why K41D-A201R remained a functional methyltransferase, and K41D-A201K did not. The MD simulations in the presence of mRNA were more revealing, where in all cases the orientations of the side chains of A201K and A201R were affected in the double mutants (Fig. 6a,b). The side chain of A201R in the double mutant pointed away from D41 (Fig. 6b). For this second conformation, observed in the presence of mRNA, the electrostatics around the side chain of residue 201 were no longer (for K41D-A201R) and less (for K41D-A201K) influenced by D41 (Fig. 6a,b and Supplementary Fig. 7). Accordingly, the electrostatics around D41 were also affected by the different conformations of the residues in position 201 in presence of mRNA (Fig. 6a,b and Supplementary Fig. 7). Upon binding in the active site, cap 0 mRNA (m7GpppGA) introduces an extra polar group (2′OH) in proximity to the active site residues, perturbing the local electrostatics. If K41 participates in mRNA binding as previously suggested^21^, the introduction of a negative charge at this position would either affect the substrate binding efficiency or would cause local conformation changes (to accommodate the extra polar group) or both. Our results suggested that mRNA binding may be affected in the presence of D41 (Fig. 5) but local conformational changes did occur too as shown in the MD simulations in the presence of mRNA.The hydrogen-bonds around D41 were thus re-analysed in presence of mRNA. Throughout the MD trajectories, D41 was hydrogen-bonded to R209 in the K41D-A201K mutant, whereas it was hydrogen-bonded to Q198 in the K41D-A201R mutant (Fig. 6c,d). In the inactive K41D-A201K variant, D41 steered the R209 side chain away from its native position, and rotated the guanidine group by 90°. This re-orientation disrupted the integrity of the active site 1st shell (Fig. 6a), potentially precluding the activation of K175. In order to sense indirect effects of the 2nd and 3rd shell mutations on the 1st shell residues, the root-mean-square deviation (RMSD) of atomic positions of the side chains of K175, D138, R209 and E207 were reported along the MD trajectories. Overall, the network formed by the first shell residues was more “rigid” (i.e. lower RMSD) in the active variants A201K and A201R, with similar median RMSDs as the WT (Fig. 7). The most flexible 1st shell was measured in the inactive K41D-A201K variant (Fig. 7). This suggested that the A201R mutation “rigidified” the first-shell residues in the K41D-A201R variant, whereas A201K did not achieve this in the inactive K41D-A201K variant (Fig. 7), providing a rationale for the difference in activity between K41D-A201K and K41D-A201R. It is possible that the different local flexibility in the active site of the methyltransferase variants, may also explain the differences in their stability.

## Discussion

None of the first-shell mutations identified in orthologous and homologous sequences yielded active methyltransferase. The only mutations that appeared to be tolerated for retaining the methyltransferase activity, were located in the third shell, corresponding to a distance of > 6 Å from the primary amine of K175. Of the six mutations tested in position 201, only basic residues were strictly tolerated in retaining the transferase activity, despite the addition of a positive charge. Interestingly, the relatively neutral mutation A201G was detrimental to the methyltransferase function. Despite being identified in the family dataset, polar residues were highly deleterious: the mutation A201Q did not yield an active transferase and the enzyme was weakly stable, suggesting the context-dependence of a functional A201Q mutation. Interestingly, the tolerated mutation A201R was found in a close ortholog (> 50% sequence identity), whereas the detrimental A201Q was identified in a more distant orthologous sequence (< 30% sequence identity), highlighting sequence-context constraints imposed at this position. Activity and stability were both affected by the mutations A201D, A201T, A201G and A201Q. By contrast, A201R was tolerated for both properties, whereas A201K was tolerated for the transferase activity but deleterious to thermostability. None of the basic mutations seemed to destabilise the integrity of the first shell (Fig. 7) or the correct positioning of the catalytic lysine (Fig. 5).

The interactions between residues D41 and K/R201 exemplify the complexity of the local fitness landscape of an enzyme’s active site, and the different effects of mutations on various features: here transferase activity and stability. The K41D mutation was tolerated in the presence of A201R for both function and stability, whereas it was deleterious to both properties in the single mutant, and deleterious only to the transferase function in the presence of A201K. MD simulations suggested different rescue mechanisms by A201R within the D41 phenotype. An efficient charge masking effect by a strong interaction between the two residues may explain the retention of stability. The indirect effect of A201R in favouring the interaction between D41 and Q198, avoided the strongly deleterious disruption of the first-shell network observed in the K41D-A201K mutant. Our results thus further confirmed the essential electrostatic role of R209^19^ for methyltransferase activity. The K-D-K motif (i.e. for VP39: K41, D138, K175) previously described as a methyltransferase catalytic triad^22^, was however not essential in one of our engineered enzyme variants. Instead, our results converged towards the essential role of the tetrad D138-K175-E207-R209. Maintaining the integrity of the tetrad (e.g. via a rescue mutation as shown herein) appeared important for the stability of the enzyme and essential for the transferase activity.

Activity and stability, two metrics of enzyme fitness tested in this study, are globally encoded in their polypeptide sequence. The fitness landscapes of both metrics do not overlap, making it extremely difficult to predict the effect of mutations. Nearly all mutations were deleterious, despite being identified in homologous, yet distant sequences, highlighting the evolutionary optimisation of the electrostatic activation of K175. These results also suggest that relatively few active-site sequence variations of VP39 retain a functional methyltransferase, rendering the engineering for higher efficiency of such enzymes a challenging task. Long distance epistatic interactions are known to influence the activity^24^ and stability^25^ of enzymes. The results presented herein suggest that targeting medium distance interactions can provide an engineering solution for reaching higher catalytic performance in mRNA methyltransferase, as illustrated by the 1.7-fold improved activity in the K41D-A201R variant. Our approach aimed at modulating active-site electrostatics, by targeting key residues with orthologous sequence options, was clearly limited by the low number of sequences in the family. However, it has revealed that functional mutations near the active site can be derived from close homologous sequences. Exploring more closely related sequences in enzyme family and/or performing systematic and extensive mutagenesis^26^, coupled to ultrahigh throughput screening^27^ and next generation sequencing, provide ways to generate a higher resolution picture of the local fitness landscape for mRNA methyltransferase active sites.

## Methods

### Cloning and mutagenesis

A codon optimised gene for *E coli* expression encoding VP39 from the *Vaccinia virus* Ankara strain (333 amino acids) was cloned into the pRSFDuet-1 (Merk-Millipore) between the *BamHI* and *EcoRI* restriction sites such that the gene was in translational fusion with a 6-His tag at the N-terminus. Site-directed mutagenesis was performed by back-to-back PCR amplifications using Forward primers bearing the mutation to introduce. After treatment by DpnI for 1 h at 37 °C (Thermofisher), a type IIs restriction enzyme (i.e. *BsaI* (Thermofisher)) was used to produce a complementary overhang after overnight digestion at 37 °C, and purification of the PCR amplicons on agarose gel. Ligation of the restricted amplicons was performed at 22 °C during 2 h using T4 DNA ligase (Thermofisher). Sanger sequencing (Source BioScience, Cambridge, UK) was used to confirm the mutations using the T7 and T7T universal primers.

### Protein expression and purification

Plasmids encoding the VP39 gene were transformed into *E coli* BL21-Gold (DE3) (Agilent) by electroporation. Single colonies were used to inoculate 10 mL Luria Bertani (LB) medium containing 40 µg mL^−1^ Kanamycin (Thermofisher) and grown overnight at 37 °C in a shaking incubator. The overnight cultures were used to inoculate in a 1/100 (v/v) dilution 50 mL LB medium containing 40 µg mL^−1^ Kanamycin (Thermofisher) in 250 mL baffled flasks. Cultures were grown for 2 h at 37 °C in a shaking incubator until an OD_600_ ~ 0.5 was reached. Protein expression was induced by isopropyl-b-D-thiogalactoside (IPTG) at a final concentration of 1 mM for 16 h at 22 °C. Cells were harvested by centrifugation and resuspended in 5 mL (10% (v/v) Nonidet P-40 (Insight Biotechnology) in 50 mM Tris–HCl pH 7.5 containing 100 mM NaCl) and lysed by sonication. The cell lysate was clarified by centrifugation (18,000 g for 15 min at 4 °C). VP39 variants were then purified using His SpinTrap columns (GE Healthcare Life Sciences) and eluted in 20 mM Tris–HCl pH 7.5 containing 0.5 M NaCl and 150 mM imidazole, before buffer exchange into 50 mM Tris–HCl pH 7.5 containing 30 mM MgCl_2_ using 10 kDa Amicon Ultra centrifugal filter units (Millipore). The purity of the preparations was assessed by SDS-PAGE using Novex 4–20% Tris–glycine gels, and protein concentrations were measured in UV-transparent cuvettes, by absorbance at 280 nm (ε = 44,810 M^-1^ cm^−1^).

### Methyltransferase activity assay

The methyltransferase activities were measured using a 2-step assay: i. *enzymatic reactions:* the VP39 variants were incubated in presence of the substrates and ii. *fluorometric assay:* the product of the enzymatic reaction was quantified using a coupled-assay producing a fluorescent product. *Enzymatic reactions:* VP39 variants (0.5–0.7 µM) were tested in the presence of s-adenosylmethionine (10 µM) and Luciferase cap 0 mRNA (4.5 µM), in 50 mM Tris–HCl pH 7.5, containing 30 mM MgCl_2_ and 25 mM ATP in a total volume of 40 µL in a 96-well plate. VP39 WT and VP39 K175C^18^ controls were used to normalise and compare VP39 variant activities. A positive control containing no enzyme, but 10 µM s-adenosylhomocysteine and 4.5 µM Luciferase cap 0 mRNA in 50 mM Tris–HCl pH 7.530 mM MgCl_2_ and 25 mM ATP, was included. Reactions were incubated for 2 h at 37 °C. *Fluorometric assay:* then, a methyltransferase fluorometric assay kit (Cayman) was used to quantify the concentration of s-adenosylhomocysteine. The kit consisted of an enzymatic cascade reaction containing s-adenosylhomocysteine nucleosidase, Adenine deaminase, Xanthine oxidase and Horseradish peroxidase leading to the formation of resorufin at a concentration proportional to the initial concentration of s-adenosylhomocysteine. 10 µL of *enzymatic reactions* were added to 100 µL of the fluorometric assay kit mixture, and fluorescence intensity (λ_excitation_ = 530 nm; λ_emission_ = 594 nm) of the produced resorufin was monitored over 30 min on a Tecan Infinite M200 Pro (Mannedorf, Switzerland). Initial rates of production of resorufin were used to determine the concentrations of s-adenosylhomocysteine in the enzymatic reactions: the initial rates were normalised by the protein concentrations in the assay and directly compared to the initial rates obtained with the samples using VP39 WT and the knock-out mutant K175C. The initial rates obtained from the positive control (5 µM s-adenosylhomocysteine—corresponding to the concentration of product in the event of all mRNA was being methylated) confirmed that none of the enzymatic reactions saturated after 2 h (Supplementary Fig. 2).

### Thermal transition measurements

Thermal transitions were determined by measuring the intrinsic fluorescence of proteins using 1 mg mL^−1^ solutions in a UNit fluorometer (Unchained Labs) that used 9 µL-micro cuvettes. Samples were excited using a 266 nm laser and the barycentric means (defined as λ_bcm_ = Σ(λ)/(Σ_λ_ Ι(λ)); where λ is the wavelength and I(λ) is the fluorescence intensity at wavelength λ) of the emission spectra were used to report on the unfolding process. The samples were first incubated 3 min at 25 °C before the temperature increased up to 90 °C following a ramp of 1 ºC.min^-1^ with an equilibration time of 30 s for each temperature. All samples were measured in triplicate. Melting temperatures (T_m_) were determined by fitting a single (I_N_ + (I_D_−I_N_)/(1 + exp((T_m1_−T)/a)); or two-transition model (I_N_ + (I_2_−I_N_)/(1 + exp((T_m1_−T)/a)) + I_2_ + (I_D_−I_2_)/(1 + exp(T_m2_−T)/b)), where I_N_ and I_D_ are the native and denatured baseline intercepts, a the cooperativity factor for the first (or only) transition, I_2_ is the baseline intercept at the intermediate conformational state, and b is the cooperativity factor for the second transition. T_m1_ corresponds to the unique thermal transition when a single transition model was used or to the first thermal transition when a two-transition model was used.

### Sequence similarity network, multiple sequence alignment and structure model of Q9EMT4

All sequences from the PFAM family PF01358 were retrieved from the EMBL-EBI Pfam database^10^. A total of 106 sequences were used in an all-versus-all BLAST (National Center for Biotechnology Information, version 2.5.0 +), considering sequence similarity only when the alignment score was below an E-value threshold of e^-30^ (corresponding to a median 32.9% sequence identity over > 250 residues). This threshold was found useful to separate sequences as a function of species (Virus versus Eukaryotes). The sequence similarity scores were imported into Cytoscape (version 3.5.1) and the network was built as previously described^21^. Multiple sequence alignments (MSA) were generated in T-Coffee^28^ using default parameters and analysed in Jalview. Three MSA were generated: 1. using the 32 sequences surrounding VP39 in the SSN (‘local’ network), 2. using the 43 sequences in the ‘extended’ square in Fig. 1c (‘extended’ network, e.g. comprising the sequence of Q9EMT4) and 3. using the 106 sequences from the PFAM P0138 family. Logo plots for the probability of the residue identities at the positions corresponding to the region of interest in VP39 in the sequences in each MSA were generated using http://weblogo.threeplusone.com/create.cgi. The structure model of Q9EMT4 was generated using Phyre2^29^ with default parameters.

### Molecular dynamics

All molecular dynamics (MD) simulations were performed using Gromacs (5.0.4)^30^. The OPLS-AA force field^31^ was used for the simulations in absence of mRNA. The 1.85 Å resolution structure of VP39 WT (PDBID: 1vp3^11^) was used and the missing residues 142–147 were modelled using Modeller 9^32^, selecting the structure with the lowest energy profile. Side-chain rotamers of the residues in the modelled loop were further optimised using Scwrl4^33^. All mutants were constructed from the same starting structure using the PyMol (Schrödinger, USA) Mutagenesis Wizard Tool. The initial structures were solvated in a cubic simulation box using the spc216 water model and Na^+^ was used to neutralise the negative charges in the system. Energy minimisation was performed using the steepest descent method (50,000 steps). Two 100-ps position-restricted simulations were performed under NVT and NPT ensembles, respectively, with all but hydrogen atoms fixed. Simulations were performed for 30-ns performed in triplicate on the whole system at 300 K. For simulations in the presence of cap 0 mRNA (m^7^GpppGA), the CHARMM force field^34^ with additions^35^ was used with a 2.8 Å structure co-crystallised in the presence of capped mRNA (PDBID: 1av6^12^). The mutant constructions, system equilibrations and simulations were performed as described above. All analysis on the trajectories was performed by extracting single frames after energy equilibration of the system suggested by a steady-state RMSD. Hydrogen-bonds between residues of interest were extracted from frames along MD trajectories using the *hbond* command in Gromacs. The command computed angles and distances between potential hydrogen atom donors and acceptors in order to define hydrogen-bonds based on the thresholds defined in Gromacs.

## Supplementary Information

Supplementary Information.
