# Supplementary material for: Functional and computational identification of a rescue mutation near the active site of an mRNA methyltransferase
Source: Sci Rep. 2020 Dec 14;10:21841. doi: 10.1038/s41598-020-79026-2 (PMC7736282; doi:10.1038/s41598-020-79026-2)
Supplement: Supplementary file 1 — Supplementary Information. [file 41598_2020_79026_MOESM1_ESM.pdf]

Supplementary Information

Functional and computational identification of a rescue mutation near the active site of an mRNA methyltransferase

Pierre-Yves Colin[1], Paul A. Dalby[1*]

[1]Department of Biochemical Engineering, University College London, London WC1H 0AH, United Kingdom
*corresponding author: Paul A. Dalby (p.dalby@ucl.ac.uk)

List of figures and tables

Supplementary Figure 1: Structural alignment of VP39 and a model structure of Q9EMT4

Supplementary Table 1: Effects of active site mutations on the methyltransferase activity and thermostability of VP39

Supplementary Table 2: Direct neighbouring residues of position 201 in the different orthologous sequences.

Supplementary Figure 2: Logo plots for the probability of the residue identities at the positions corresponding to the region of interest in VP39 in close homologues and viral homologs.

Supplementary Figure 3: Quantification of s-adenosylhomocysteine using a fluorometric assay allows the measurement of the relative activity of the VP39 variants.

Supplementary Figure 4: "Acceptability" of orthologous mutations as a function of the percentage of identity between orthologous sequences and VP39.

Supplementary Figure 5: Annotated SDS-PAGE gels of purified VP39 WT and all the active site variants in this study

Supplementary Figure 6: Correlation between melting temperature (T_{m1}) and red-shift values

Supplementary Figure 7: Interaction between residues 41 and 201 revealed by pKa analysis

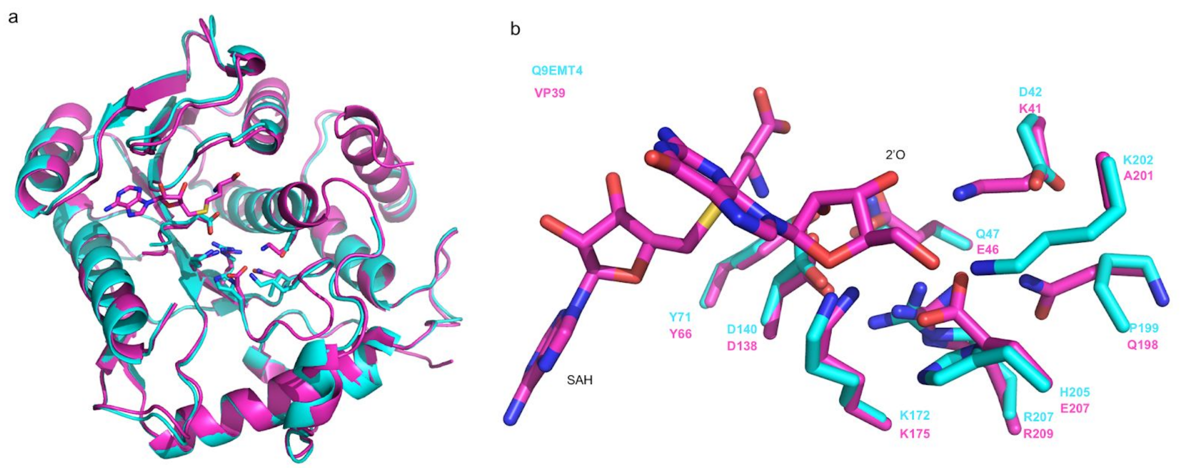

Supplementary Figure 1: Structural alignment of VP39 and a model structure of Q9EMT4

Superimposition of the crystal structure of VP39 (PDBID:1av6) and a model structure of *Amsacta moorei entomopoxvirus* (Q9EMT4). a. Overall fold superimposition of the two structures. b. Superimposition of the active site residues of VP39 (1av6, in pink) and Q9EMT4 (in cyan).

Supplementary Table 1: Effects of active site mutations on the methyltransferase activity and thermostability of VP39

The methyltransferase activity is normalised to the activity measured with the WT enzyme. s.d.1 values correspond to the standard deviations between all the activity measurements. (T_{m1}) in the melting curve measured by protein intrinsic fluorescence. ΔT_{m1} reports the difference in T_{m1} between the mutant and the WT proteins. All measurements were performed in triplicates at a concentration of 1 mg/mL. s.d.2 values correspond to the standard deviations between the T_{m1} obtained from fitting single or double transition models in the three replicates.

**Variant**	**Methyltransferase Activity**		**Thermostability**		
	normalised to WT	s.d. 1	T_{m1}	s.d. 2	ΔT_{m1} (ºC)
K41A	-	-	58.5	0.2	-2
K41D	0.0	0.2	53.4	0.2	-7
K41E	-	-	54.8	0.2	-6
D138E	-	-	57.4	0.1	-3
K175C	0.0	0.1	60.2	0.1	0
A201D	0.2	0.4	54.5	0.1	-6
A201G	0.5	0.4	57.7	0.2	-3
A201K	1.0	0.4	51.4	0.2	-9
A201Q	-	-	47.5	0.2	-13
A201R	1.2	0.3	59.9	0.1	-1
A201T	0.6	0.2	57.4	0.1	-3
E207H	-	-	51.1	0.1	-9
R209Q	0.1	0.2	54.4	0.3	-6
WT	1.0	0.3	60.5	0.2	0
K41D-A201K	-	-	50.2	0.1	-10
K41D-A201R	1.7	0.5	60.1	0.0	0
K41D-E207H	-	0.2	48.9	0.1	-12
E46Q-A201K	0.2	0.2	53.8	0.8	-7
E46Q-A201R	0.2	0.3	60.5	0.0	0
A201K-E207H	-	-	44.6	0.2	-16

A201R-E207H	-	-	51.5	0.2	-9
K41D-A201K-E207H	-	-	46.7	0.2	-14
K41D-A201R-E207H	-	-	51.5	0.1	-9

Supplementary Table 2: Direct neighbouring residues of position 201 in the different orthologous sequences.

The positions were identified from a multiple sequence alignment using all sequences from the PFAM PF01358 as input and using the T-Coffee algorithm with default parameters. Residue numbers refer to the VP39 sequence.

Residue number	37	41	198	199	200	201	202	205	244
VP39	Q	K	Q	P	F	A	P	S	R
P07617	Q	K	Q	P	F	A	P	S	R
P15916	Q	K	Q	P	F	A	P	S	R
P33052	Q	K	Q	P	F	A	P	S	R
P68544	Q	K	Q	P	F	A	P	S	R
A0A061J2G0	F	K	G	V	W	A	P	S	R
A0A0M9FZ82	Y	K	G	V	W	A	P	S	R
A0A0N1HV56	Y	K	G	V	W	A	P	S	R
A0DNV8	W	K	Q	A	W	A	P	S	R
A0E8V4	W	K	Q	A	W	A	P	S	R
A4HD69	Y	K	G	V	W	A	P	S	R
E9AH23	Y	K	G	V	W	A	P	S	R
K2MVF4	F	K	G	V	W	A	P	S	R
Q08FS5	Q	K	Q	P	F	A	P	S	R
Q385S9	Y	K	G	V	W	A	P	S	R
Q4CYZ2	F	K	G	V	W	A	P	S	R
Q4DRV3	F	K	G	V	W	A	P	S	R
Q4QAW9	Y	K	G	V	W	A	P	S	R
Q6TUU7	Q	K	Q	P	F	A	P	S	R
Q6TVR7	R	K	Q	P	F	A	P	S	R
Q8V3N0	Q	K	Q	P	F	A	P	S	R
Q98244	Q	K	Q	P	F	A	P	S	R
Q9Q906	Q	K	Q	P	F	A	P	S	R

S9UQZ5	Y	K	G	V	W	A	P	S	R
S9URG3	Y	K	G	V	W	A	P	S	R
S9UYJ0	Y	K	G	V	W	A	P	S	R
S9V7U5	Y	K	G	V	W	A	P	S	R
S9VDE1	Y	K	G	V	W	A	P	S	R
S9VVJ7	Y	K	G	V	W	A	P	S	R
V5BRC4	F	K	G	V	W	A	P	S	R
W6KLL2	F	K	G	V	W	A	P	S	R
A0A0D2UAZ 4	W	K	P	V	W	G	P	T	R
A0A0D2WKF 8	W	K	P	V	W	G	P	T	R
A9V270	W	K	P	I	W	G	P	T	R
C5KXU9	W	K	P	I	W	G	A	T	R
D2VM22	W	K	P	V	W	G	R	T	R
D2VMH7	W	K	P	V	W	G	P	T	R
F2TYB8	W	K	P	V	W	G	P	T	R
F2U5G7	W	K	P	C	W	G	G	T	R
Q9EMY9	Q	K	Q	C	F	Q	K	S	R
Q070F5	Q	K	Q	P	F	R	P	S	R
A0A0D2WM G3	W	K	P	V	W	T	G	S	R
A0A0L1KNW 2	W	K	P	L	W	T	R	S	R
A0A0M9GA W4	W	K	P	I	W	T	R	S	R
A0A0N1PB31	W	K	P	I	W	T	R	S	R
A4HN05	W	K	P	I	W	T	R	S	R
A4IBM8	W	K	P	I	W	T	R	S	R
E9AFG2	W	K	P	I	W	T	R	S	R
K2N176	W	K	P	I	W	T	R	S	R
Q38DJ3	W	K	P	I	W	T	R	S	R

Q4DU96	W	K	P	I	W	T	R	S	R
S9TZF9	W	K	P	I	W	T	R	S	R
S9UQ66	W	K	P	I	W	T	R	S	R
S9VLV7	W	K	P	I	W	T	R	S	R
V5BTE5	W	K	P	I	W	T	R	S	G
W6KM83	W	K	P	I	W	T	R	S	R

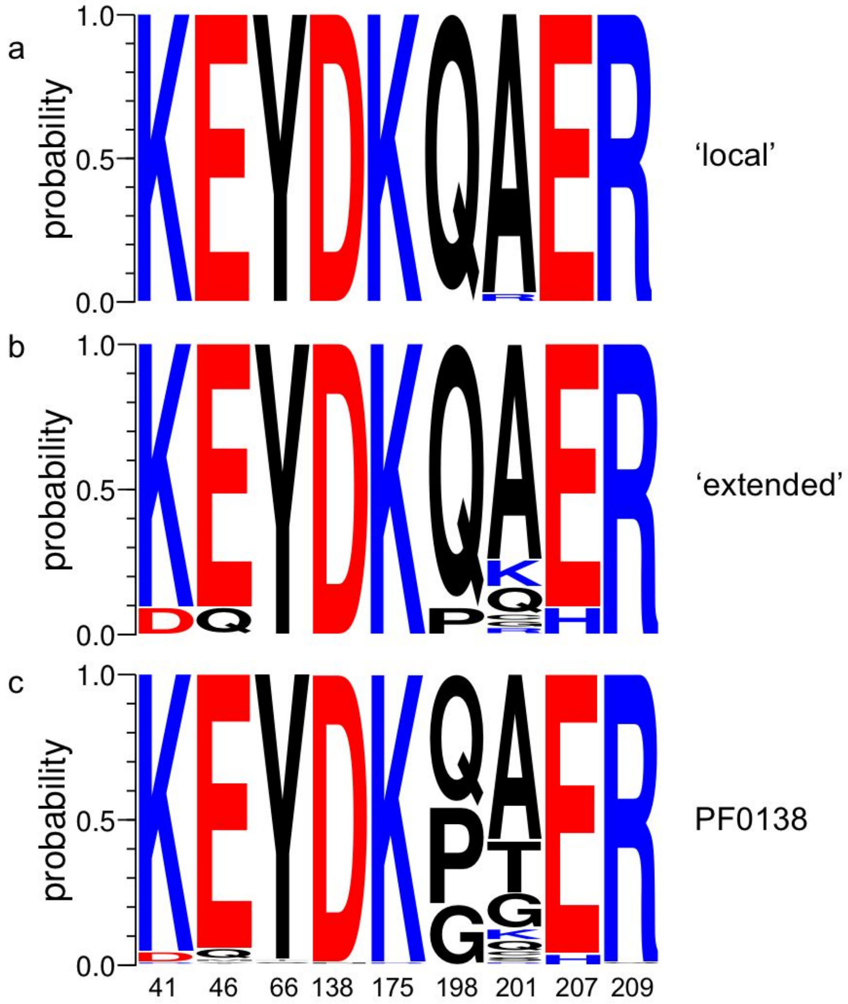

Supplementary Figure 2: Logo plots for the probability of the residue identities at the positions corresponding to the region of interest in VP39 in close homologs, viral orthologs and in the PF0138 family.

The positions correspond to the residues K41, E46, Y66, D138, K175, Q198, A201, E207 and R209 in VP39. a. The 'local' probability logo plot was calculated using a multiple sequence alignment of the 32 direct neighbours of VP39 indicated by the 'local' square in the sequence similarity network (Figure 1c of the main manuscript). b. The 'extended' probability logo plot was calculated using a multiple sequence alignment of the 42 sequences indicated by the 'extended' square in the sequence similarity network (Figure 1c of the main manuscript). These sequences constitute a connected network of sequences in the SSN, i.e. each sequence shares at least 32.9% sequence identity over 250 residues with at least 1 other member of the network. c. The PF0138 probability logo plot was calculated using a multiple sequence alignment of the 106 sequences in the PF0138 PFAM family.

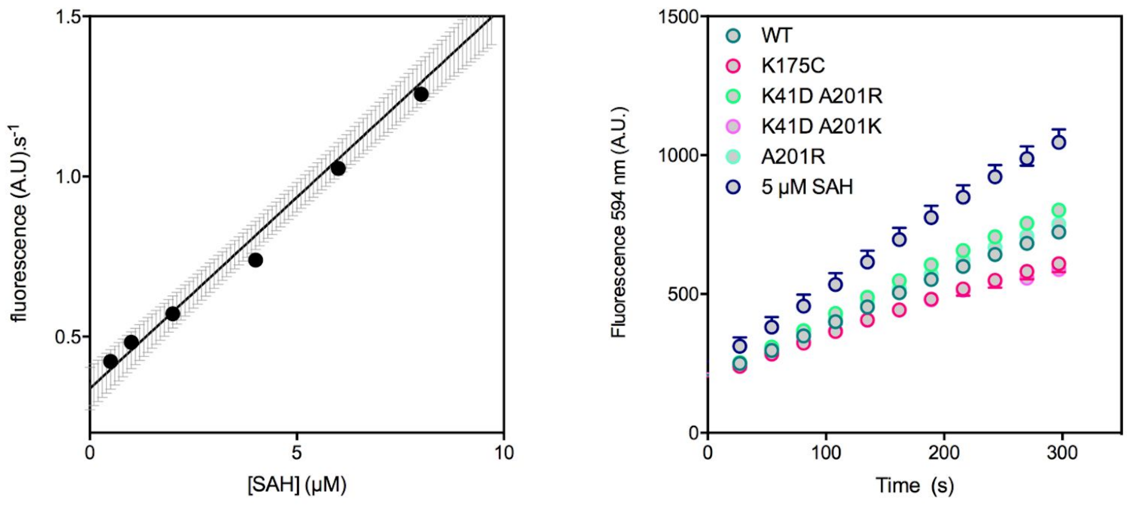

Supplementary Figure 3: Quantification of s-adenosylhomocysteine using a fluorometric assay allows the measurement of the relative methyltransferase activity of the VP39 variants.

a. Initial rates of resorufin production as a function of s-adenosylhomocysteine concentrations. The correspondence between the concentration of s-adenosylhomocysteine (SAH) and the fluorescence signal (measured as described in the Method of the main manuscript) was found to be linear within the relevant range of SAH concentrations of the enzymatic assay. Error bars correspond to the 95% confidence of the linear regression fit.

b. Examples of initial rates of resorufin production in the fluorometric assay for five VP39 variants and the positive control '5μM SAH'. Each point represents the median value of three technical replicates and the bars correspond to the range of values.

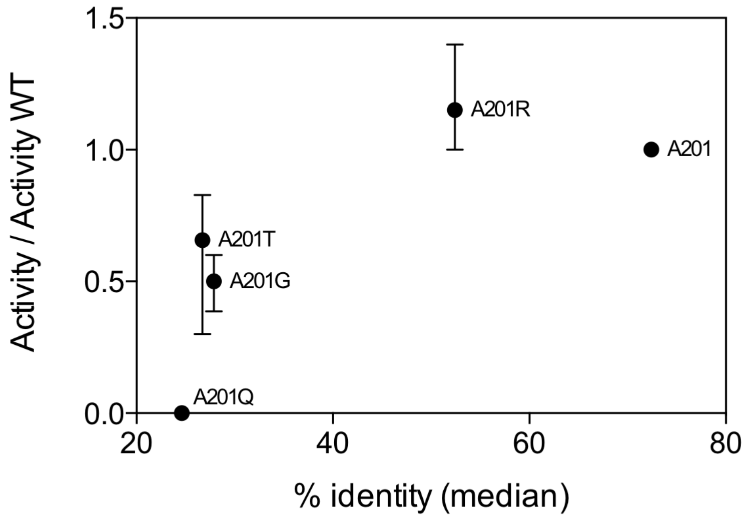

Supplementary Figure 4: "Acceptability" of orthologous mutations as a function of the percentage of identity between orthologous sequences and VP39.
The median percentage of identity was calculated from 13 sequences for A201 and 5 sequences for A201T. A201G was only found in two sequences whereas only one orthologous sequence bore the mutations A201R or A201Q respectively in the dataset PFAM PF01358. The activity ratios correspond to the data shown in Table 1 of the main manuscript.

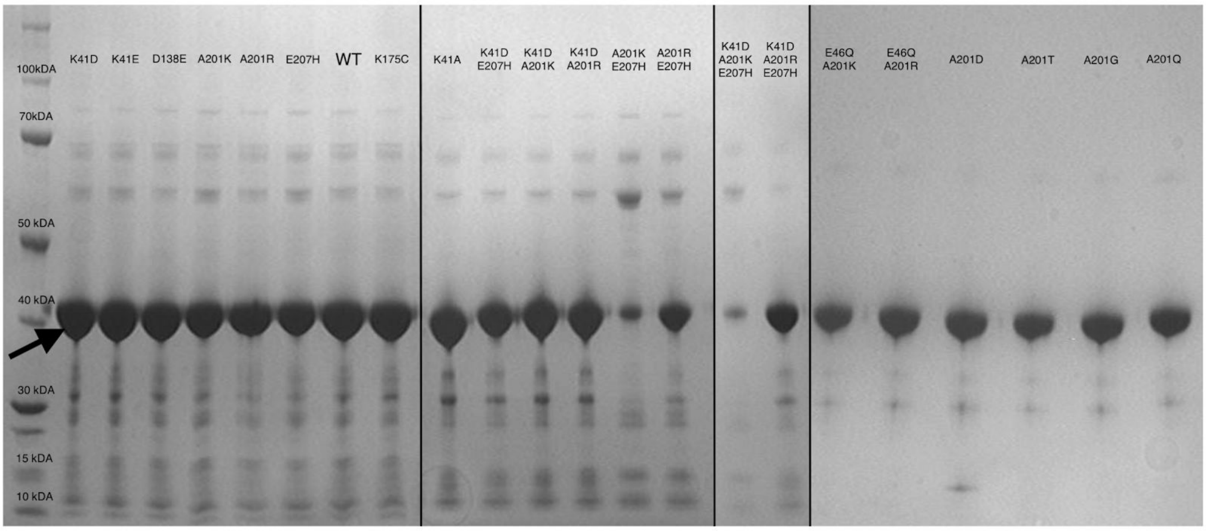

Supplementary Figure 5: Annotated SDS-PAGE gels of purified VP39 WT and all the active site variants in this study

From left to right samples are: Ladder (PageRuler^{TM} unstained broad range protein), K41D, K41E, D138E, A201K, A201R, E207H, WT, K175C, K41A, K41D-E207H, K41D-A201K, K41D-A201R, A201K-E207H, A201R-E207H, K41D-A201K-E207H, K41D-A201R-E207H, E46Q-A201K, E46Q-A201R, A201D, A201T, A201G, A201Q. The image results from 4 gels stitched together. The black arrow indicates the bands of interest corresponding to the N-terminal His-tagged VP39 variants.

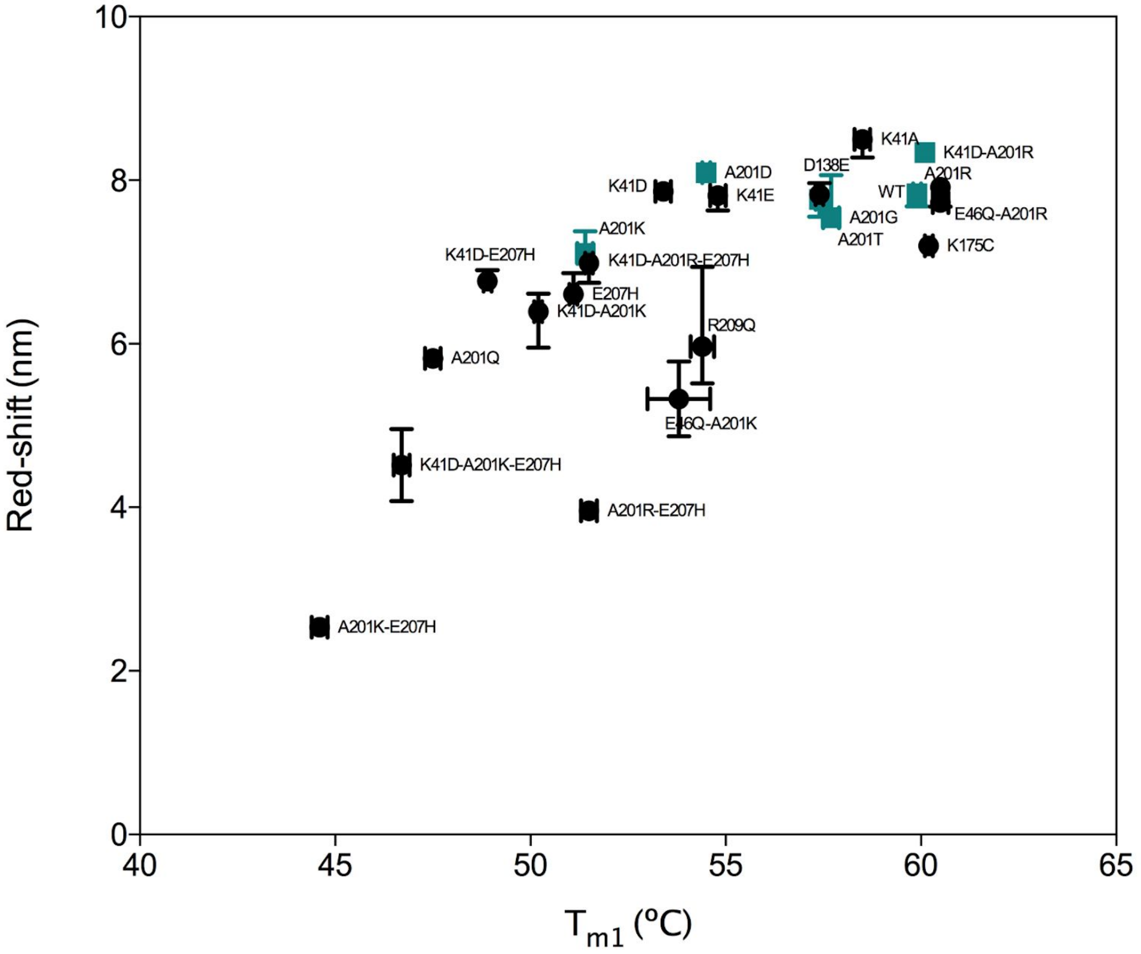

Supplementary Figure 6: Correlation between melting temperature (T_{m1}) and red-shift values in all methyltransferase variants tested in this study.

T_{m1} represents the first thermal transition upon protein unfolding and red-shift is the difference between the tryptophan fluorescence barycentric means of the folded and unfolded states of each protein variant. Variants with detectable methyltrasnferase are represented by green squares, other variants represented by black squares have no detectable methyltransferase activity.

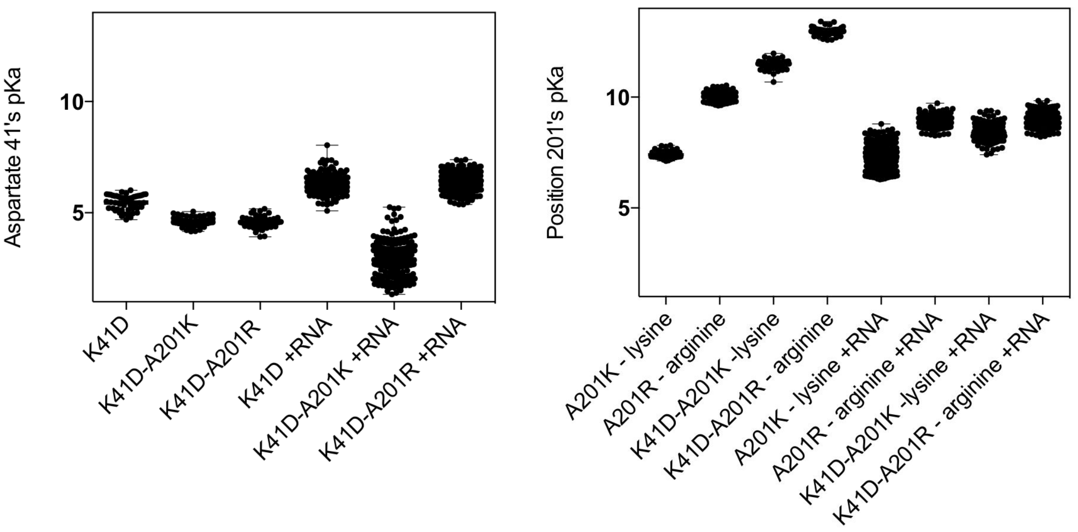

Supplementary Figure 7: pKa values of epistastic residues in the third shell of the active site in presence or absence of mRNA.

pKa values were calculated using propka 3.0 from frames extracted every 20 or 100 ps along 10 to 25 ns molecular dynamics trajectories.

Aspartate 41's pKa was not influenced by the presence of a lysine or an arginine at position 201, however, RNA did shift D41's pKa in presence of RNA in the A201K background. The K41D increased the pKa of both lysine and arginine at position 201, in absence of RNA. In the presence of RNA, the electrostatic effects of K41D on 201K and 201R were not detected.
